# Water Relations and Foliar Isotopic Composition of *Prosopis tamarugo* Phil., an Endemic Tree of the Atacama Desert Growing at Three Levels of Water Table Depth

**DOI:** 10.3389/fpls.2016.00375

**Published:** 2016-03-30

**Authors:** Marco Garrido, Paola Silva, Edmundo Acevedo

**Affiliations:** ^1^Programa de Doctorado en Ciencias Silvoagropecuarias y Veterinarias, Universidad de ChileSantiago, Chile; ^2^Soil-Plant-Water Relations Laboratory, Agricultural Production Department, Faculty of Agronomical Sciences, University of ChileSantiago, Chile

**Keywords:** ^13^C isotopic composition, ^18^O isotopic composition, phreatophyte, stomatal behavior, water table depth

## Abstract

*Prosopis tamarugo* Phil. is a strict phreatophyte tree species endemic to the “Pampa del Tamarugal”, Atacama Desert. The extraction of water for various uses has increased the depth of the water table in the Pampa aquifers threatening its conservation. This study aimed to determine the effect of the groundwater table depth on the water relations of *P. tamarugo* and to present thresholds of groundwater depth (GWD) that can be used in the groundwater management of the *P. tamarugo* ecosystem. Three levels of GWD, 11.2 ± 0.3 m, 10.3 ± 0.3 m, and 7.1 ± 0.1 m, (the last GWD being our reference) were selected and groups of four individuals per GWD were studied in the months of January and July of the years 2011 through 2014. When the water table depth exceeded 10 m, *P. tamarugo* had lower pre-dawn and mid-day water potential but no differences were observed in minimum leaf stomatal resistance when compared to the condition of 7.1 m GWD; the leaf tissue increased its δ^13^C and δ^18^O composition. Furthermore, a smaller green canopy fraction of the trees and increased foliage loss in winter with increasing water table depth was observed. The differences observed in the physiological behavior of *P. tamarugo* trees, attributable to the ground water depth; show that increasing the depth of the water table from 7 to 11 m significantly affects the water status of *P. tamarugo*. The results indicate that *P. tamarugo* has an anisohydric stomatal behavior and that given a reduction in water supply it regulates the water demand via foliage loss. The growth and leaf physiological activities are highly sensitive to GWD. The foliage loss appears to prevent the trees from reaching water potentials leading to complete loss of hydraulic functionality by cavitation. The balance achieved between water supply and demand was reflected in the low variation of the water potential and of the variables related to gas exchange over time for a given GWD. This acclimation capacity of *P. tamarugo* after experiencing increases in GWD has great value for the implementation of conservation strategies. The thresholds presented in this paper should prove useful for conservation purposes of this unique species.

## Introduction

The mortality rate of forest communities in different biomes has experienced substantial increases due to higher temperatures and incidence of more frequent and severe droughts (Peng et al., 2011; Liu et al., 2013; Williams et al., 2013). In addition, the disturbance of the environment by human activity, such as pollution or mining, exerts a substantial control over the species composition and structure of the forests, either by itself or by enhancing the effects of a stressor (Frelich, 2002). Whatever the case, the impact of natural stress or disturbance on plant species generates damage at various levels, from the photosynthetic activity to the survival capacity of the individual, the extent of the damage being dependent on the intensity of the phenomenon and the plant resistance to the stress (Thomas and Packham, 2007).

Human economic advance has caused high pressure on the use of natural resources. An example of this is the removal of water from the deserts for its use in urban centers and industrial activities (Pringle, 2001) endangering the species that live there. Such is the case of the Pampa del Tamarugal and Llamara aquifer, ecosystem having a hyper-arid climate (Pliscoff and Leubert, 2006), located in northern Chile in the Atacama Desert.

The vegetation of the Llamara Salt Flat is a tropical thorn forest composed by tamarugo (*Prosopis tamarugo*) and retama (*Tessaria absinthioides*). The shrub layer is composed mainly by retama (*T. absinthioides*; *Caesalpinia aphylla*), cachiyuyo (*Atriplex atacamentis*), algarrobilla (*Prosopis strombulifera*), and mesquite (*Prosopis burkartii*), while the herbaceous layer is almost completely dominated by salt grass (*Distichlis spicata*; Tréllez et al., 2011). The vegetation in the study site is composed almost entirely of *P. tamarugo* and some individuals of *T. absithioides*.

In this environment, the extraction of groundwater for the supply of human communities and mining (Rojas and Dassargues, 2007) has led to a drop in the water table out of which plant species live. The most abundant species in this ecosystem is *P. tamarugo* Phil., an endemic, strict phreatophyte legume tree (Altamirano, 2006), highly adapted to the conditions of high temperature and radiation of the Pampa del Tamarugal (Lehner et al., 2001) and described as a water stress resistant species (Acevedo et al., 1985). Water stress strongly increases the rate of defoliation of *P. tamarugo* under controlled conditions, decreases its paraheliotropic capacity (Chávez et al., 2013a), and its photosynthetic rate (Delatorre et al., 2008). Field studies in mature trees have shown that the effect of lowering the water table, increases the variability among individual trees of *P. tamarugo* (Altamirano, 1994), decreases its water potential, and causes loss of foliage (Chávez et al., 2013b), decreases NDVI (Normalized Difference Vegetation Index) as canopy activity indicator (Ortiz et al., 2012; Chávez et al., 2014) and eventually there is loss of paraheliotropic ability (Chávez et al., 2014).

A theoretical framework has been proposed in which the mortality of woody species to water stress would be governed by three factors (McDowell et al., 2008): (i) biotic agents, which are a constant factor that can intensify by the incidence of stress, (ii) hydraulic failure, which plays an important role in the mortality of leaves, shoots, and peripheral roots under water stress (Rice et al., 2004; Hoffmann et al., 2011), and (iii) carbon starvation as the depletion of the plant reserves, reducing its resilience and increasing susceptibility to other stresses (Bigler et al., 2006) such as oxidative stress (Sage and Kubien, 2007; Delatorre et al., 2008). The predominant mechanism depends on the nature of drought, its intensity, and duration (McDowell et al., 2008; McDowell, 2011) and stomatal behavior of each species (Tardieu and Simonneau, 1988). It is expected that species having isohydric stomatal regulation possess an hydraulic system more succeptible to cavitation when compared to anisohydric species (Vogt, 2001; McDowell et al., 2008; Mitchell et al., 2013), the former being associated to regions of moderate humidity, and the latter to arid zone communities (Maherali et al., 2004).

Stomatal regulation and carbon assimilation can be studied through the leaf carbon and oxygen isotopic composition; the carbon isotopic composition (δ^13^C) of the leaf tissue can be used as a proxy for carbon assimilation (the more negative its value, the higher is the C assimilation in a given period of time), and the oxygen isotopic composition (δ^18^O) of the leaf tissue can be used as an indicator of stomatal resistance integrated over a period of time, when the leaf to air vapor pressure deficit is similar across the study area (the higher the oxygen isotopic composition, the higher is the stomatal resistance; Farquhar et al., 2007); further, the ratio of these two values may be used as a proxy for the intrinsic water use efficiency (assimilation per unit of stomatal conductance; Farquhar et al., 1989). Combined analyses of the carbon and oxygen isotopic composition of leaf biomass provide a means to distinguish the separate effects of stomatal conductance and net photosynthesis on water use efficiency (Scheidegger et al., 2000). The conceptual model of Scheidegger et al. (2000) has been successfully applied in the field (Keitel et al., 2003; Sullivan and Welker, 2007), so the dual-isotope approach has proven a valuable concept for ecological applications.

The objective of this study is to provide physiological information about how tamarugo trees behave as the GWD is increasing. This information should prove useful for the management of the aquifers where *P. tamarugo* grows and for the conservation of this unique species. In the process, we use integrative variables such as carbon and oxygen isotopic composition of the leaf tissue as indicators of gas exchange and the fraction of green canopy as indicator of defoliation. The advantage of using this type of variables has already been established (Farquhar and Lloyd, 1993; Brugnoli and Farquhar, 2000; Chávez et al., 2014).

## Materials and methods

### Site characterization and monitoring

The study site is located south of the Pampa del Tamarugal, Atacama Desert, northern Chile, in the Llamara Salt Flat (19K 434222.0 m E, 7658495.0 m S) dominated by the Llamara aquifer, located in the mid depression of the Tarapaca Region. This confined aquifer has 30 to 40 Km width at a mean altitude of 1000 m (DICTUC, 2007). The Pampa del Tamarugal has a hyper-desertic tropical bio-climate (Pliscoff and Leubert, 2006) characterized by the almost complete absence of rainfall (long term mean of 5 mm year^−1^), low relative humidity, and wide temperature variation (Campillo and Hojas, 1975). Table 1 shows meteorological information obtained at the Canchones Experiment Station, Arturo Prat University, commune of Pozo al Monte, Tamarugal Province (19K 444490.5 m E; 7739128.5 m S). Rainfall during the study period was near zero.

**Table 1:** **Meteorological data from a meteorological station for the time of measurements (see text), mean monthly values for daily mean temperature, relative humidity, and wind speed; maximum solar radiation in the month and total rainfall**.

	**Meteolorogical variable**	**Year**				**Mean ± SD**
		**2011**	**2012**	**2013**	**2014**	
**JANUARY**						
	Temperature (°C)	20.5	21.1	21.4	21.5	21.1 ± 0.4
	Relative Humidity (%)	44.0	48.3	43.0	50.6	46.5 ± 3.6
	Wind speed (ms^−1^)	1.1	1.2	1.1	1.1	1.1 ± 0
	Maximum solar radiation (Wm^−2^)	1059.2	1014.5	1067.8	1050.3	1047.9 ± 23.4
	Rainfall (mm)^*^	3.4	0.8	0.0	0.0	1.1 ± 1.6
**JULY**						
	Temperature (°C)	13.7	16.1	14.4	12.9	14.3 ± 1.4
	Relative Humidity (%)	45.4	27.3	32.2	32.5	34.3 ± 7.8
	Wind speed (ms^−1^)	0.6	0.3	0.5	0.3	0.4 ± 0.1
	Maximum solar radiation (Wm^−2^)	704.9	685.6	745.2	795.6	732.8 ± 48.6
	Rainfall (mm)^**^	0.4	0.4	0.2	0.0	0.25 ± 0.2

Canchones Experiment Station, commune of Pozo al Monte, Tamarugal Province. (19K 444490.5 m E; 7739128.5 m S). Rainfall during this period was 5.2 mm, practically nil.

* *Amount of rainfall between January and June*.

** *Amount of rainfall between July and December*.

In the Llamara Salt Flat site there is a gradient of groundwater depth (GWD) caused by the presence of water extraction wells that generate a depression cone with a radius of about 4 km. After 5 years of the onset of water extraction (2006–2010), the water table depth was stabilized, and three zones of GWD were established in a linear transect from the extraction wells: (i) High-GWD (11.2 ± 0.3 m), (ii) Mid-GWD (10.3 ± 0.3 m), and (iii) Low-GWD (7.1 ± 0.1 m). The 7.1 m was used as reference GWD since previous studies covering the period 2005–2011 and GWDs from 4.0 m to 6.0–7.0 m (Calderón et al., 2015) had shown that except for a small decrease in twig growth rate, the physiological parameters studied were not affected at 7.1 m GWD. Four *P. tamarugo* trees per GWD zone were selected for the study (total of 12 trees). The depth of the water table at each tree was estimated through spacial interpolation, using actual GWDs obtained from observation wells located in the area. The depth of the water table in the observation wells was measured with a water level indicator (Waterline, USA). Table 2 shows the mean and standard deviation of the GWD for the four *P. tamarugo* trees of each group over the 4 years of this study (January 2011 through July 2014). Notably, the groundwater depth at each GWD zone has been stable over the 4 years of study. Water extraction began in February 2006 and the GWD prior to water extraction is indicated by the value of December 2005 in Table 2.

**Table 2:** **Estimated mean water table depths ± SD in a transect away from water extraction wells at the Llamara Salt Flat, Pampa del Tamarugal, Atacama Desert**.

	**Mean depth of the water table (m)**		
**Measuring date**	**High-GWD**	**Mid-GWD**	**Low-GWD**
Dec-05^*^	4.0 ± 0.1	4.3 ± 0.1	5.9 ± 0.5
Jan-11	11.1 ± 0.0	10.4 ± 0.3	7.0 ± 0.2
Jul-11	10.5 ± 0.2	9.8 ± 0.2	7.0 ± 0.2
Jan-12	11.4 ± 0.0	10.5 ± 0.5	7.1 ± 0.3
Jul-12	11.1 ± 0.2	10.2 ± 0.3	7.0 ± 0.2
Jan-13	11.3 ± 0.2	10.2 ± 0.4	7.1 ± 0.2
Jul-13	11.6 ± 0.1	10.4 ± 0.6	7.2 ± 0.2
Jan-14	11.6 ± 0.1	10.6 ± 0.5	7.3 ± 0.2
Jul-14	11.3 ± 0.2	10.5 ± 0.3	7.3 ± 0.2
Mean between Jan-11 and Jul-14	11.2 ± 0.3	10.3 ± 0.3	7.1 ± 0.1
Δ between Dec-05 and Jul-14	7.3	6.2	1.4

Pumping started in December 2005. High GWD (11.2 m deep, 150 m away from the water extraction well); Mid-GWD (10.3 m deep, 300 m away from the water extraction well); and Low-GWD (7.1 m deep, reference GWD, 3000 m away from the extraction well). Data collected from January 2011 through July 2014.

* Water table depth prior to water extraction.

The data were collected in the months of January (summer) and July (winter). The group of trees associated with the High-GWD zone was at approximately 150 m away from the water extraction wells, those associated with the Mid-GWD zone were around 300 m away and those in the Low-GWD zone were at about 3000 m away from the water extraction wells. All the trees of this study are on the Llamara aquifer of the Llamara Salt Flat, the most distant two trees are 3500 m away from the water extraction wells.

### Plant water status: Water potential and stomatal resistance

The water potential and the stomatal resistance were measured in each field campaign. The water potential was measured at pre-dawn (Ψ_pd_; around 6:00 h in summer and around 7:00 h in winter, dawn in the area is 6:44 h in January and 7:50 h in July) and around solar noon (mid-day water potential; Ψ_md_) between 12:30 and 14:30 h using a Scholander pressure chamber (Scholander et al., 1965). The twigs used for water potential determination were lignified, one season twigs which were cut at the base. Two twigs per tree which had been covered with a plastic bag and foil the previous evening were used. The mid-day water potential was measured only in the trees of the High-GWD and Low-GWD zones.

The stomatal resistance was measured between 8:30 and 10:30 h, time during which the stomata have their maximum aperture in these trees (Calderón et al., 2015). The stomatal resistance (*r*_*s*_) was measured in four fully expanded, nutritionally, and sanitary healthy leaves per tree, fully exposed to the sun (facing east). A steady-state diffusion porometer (DECAGON Devices, Steady State Diffusion Leaf Porometer Model SC-1) was used. The measurements of water potential and stomatal resistance were done the same day in the same trees.

### Soil water content

In each campaign soil samples at 20, 30, 40, 60, and 100 cm depth were taken to determine the soil water content gravimetrically. The samples were taken under the canopy of one selected tree in each GWD zone. The samples were placed in aluminum tins, weighed to determine wet weight, and dried at 105°C to determine the weight of dry soil. The soil bulk density for each depth was determined using the cylinder method (Allen, 1989), and the soil water content was expressed as a volume fraction (cm^3^ cm^−3^). Finally, weighted mean soil water (weighted per soil depth, WWC) was calculated for each 1 m soil profile.

### Leaf sampling and measurement of δ^13^C and δ^18^O composition

In the campaigns of January of each year a composite sample of leaves per each tree was taken. Sampling was done by taking leaves of the present growing season in the entire perimeter of each tree at about 1.5 m height. The size of each sample was ~50 g. Each sample was dried at 60°C in a forced air oven for 48 h. The samples were then crushed to a homogeneous powder with a power mill. Two sub-samples were taken from each sample and put in tin and silver capsules to measure isotopic composition of ^13^C (δ^13^C) and ^18^O (δ^18^O), respectively. Finally, the samples were sent to the Stable Isotope Facility at the University of California, Davis, for analysis (data available in Supplementary Material; Table 1). The samples for δ^13^C were measured using a PDZ Europa ANCA-GSL elemental analyzer interfaced to a PDZ Europa 20–20 isotope ratio mass spectrometer (Sercon Ltd., Cheshire, UK) with a precision of 0.2‰. The samples for δ^18^O were measured with an Elementar PyroCube (Elementar Analysensysteme GmbH, Hanau, Germany) interfaced to a PDZ Europa 20–20 isotope ratio mass spectrometer (Sercon Ltd., Cheshire, UK), with a precision of 0.5‰.The standards used to measure the δ^13^C isotopic composition were USGS-41 (Glutamic Acid) and Nylon 5 as international standards and Glutamic Acid and Peach leaves as the internal check. For the measurement of the δ^18^O isotopic composition the international standards were IAEA-600 (Caffeine) and USGS-35 (Sodium Nitrate) and Cellulose as an internal check.

### Green canopy fraction

The green canopy fraction (ƒGCC), used to quantitatively assess defoliation, was defined as the green surface of a side of a *P. tamarugo* tree respect to the total area of the same side and was determined according to the methodology described in Chávez et al. (2013b). In each campaign a 10 mega pixels picture of each tree was taken with a digital camera (FUJIFILM Model FINEPIX S2000HD) in automatic mode and from the same point each time. The photographs were processed with the sub-routine for image analysis based on objects of the program eCognition® Developer. Objects were identified within each image with a scale parameter of 3.0, a shape factor of 0.1, and compactness of 0.5. Then, the objects associated with “leaves” and “branches” were identified through an analysis of the green, red, and blue bands of each pixel that made up the picture. The area of “leaves” and “branches” was determined according to the number of pixels. The ƒGCC was calculated as follows,

(1) $$ƒ\text{GCC}=\frac{\text{Leaf area}}{\text{Leaf area}+\text{Branch area}}$$

In order to assess the magnitude of change in ƒGCC, each year between January and July the relative difference of green coverage from January to July (RƒGCC_Jan−Jul_) was calculated as follows,

(2) $$\text{R}ƒ{\text{GCC}}_{\text{Jan-Jul}}=\frac{ƒ{\text{GCC}}_{\text{Jan}}-ƒ{\text{GCC}}_{\text{Jul}}}{ƒ{\text{GCC}}_{\text{Jan}}}$$

### Data analysis

Statistical analyses were performed using the statistical software InfoStat (Di Rienzo et al., 2015) and/or R version 3.2.1 (R Core Team, 2015). The significance level was set at 0.05 for all comparisons. The variables ƒGCC, Ψ_pd_, Ψ_md_, *r*_*s*_, δ^13^C, δ^18^O, and RƒGCC_Jan−Jul_ were analyzed using general linear and mixed models for repeated measures, with the measuring date, GWD zone and the interaction between factors as fixed factors, and we modeled the correlation with an autocorrelation structure of order 1 (corAR1) for the measurements over the same individual (tree) between different times (measurement date), followed by LSD -Fisher *post-hoc* analyzes when appropriate (Supplementary Material; Table 2). The model selection in each case was based on the fulfillment of the assumptions of the statistical analysis and under AIC index values (Akaike information criterion; Bozdogan, 2000). The only variable that had to be transformed to meet the statistical assumptions for analysis was stomatal resistance.

## Results

### Effect of the depth of the water table on the water potential and gas exchange of *P. tamarugo*

**Figure 1:**
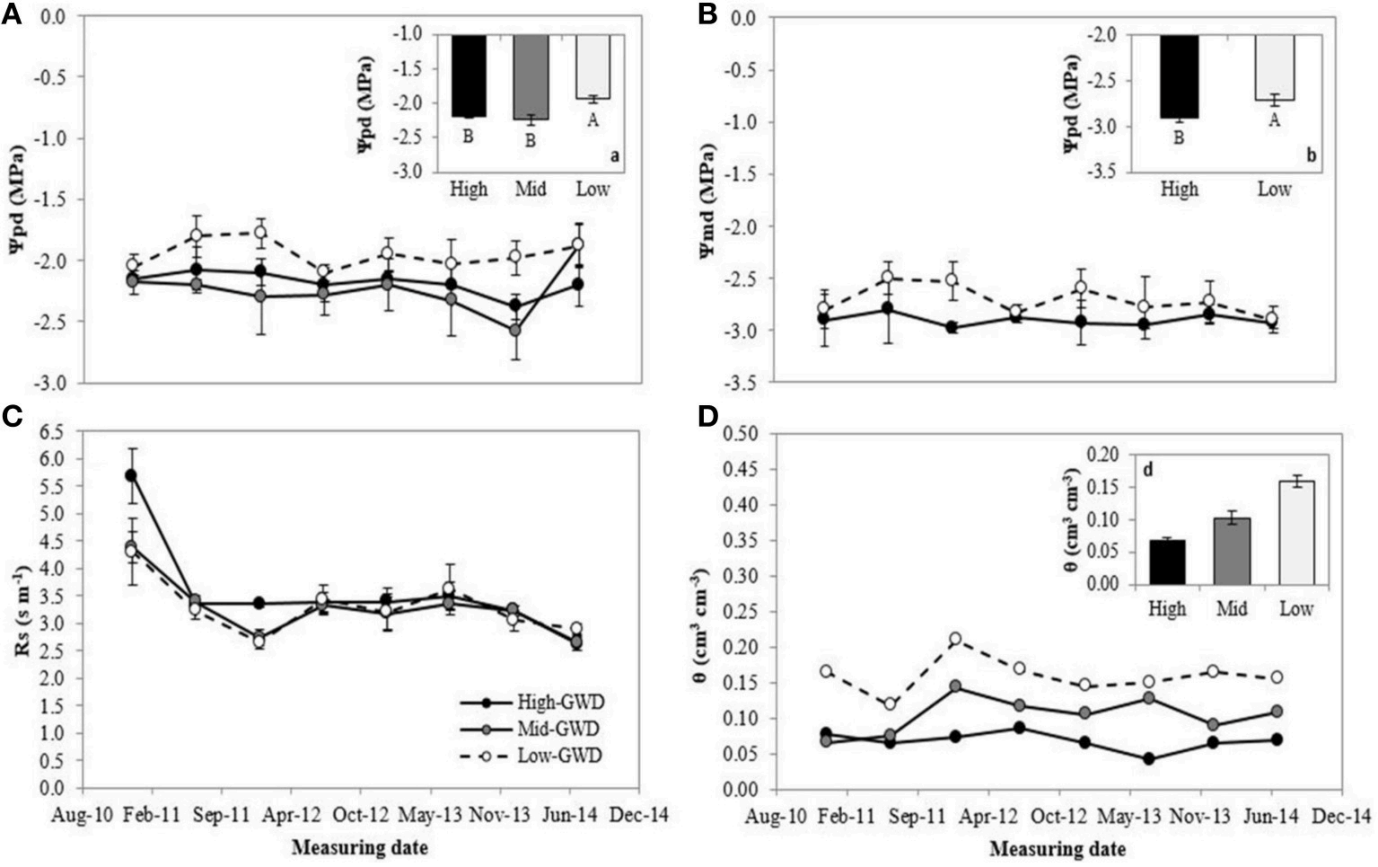
**Means ± 1 S.E. of (A) predawn water potential, (B) mid-day water potential, (C) stomatal resistance, and (D) weighted mean of soil water content**. Each curve within a graph represents a groundwater depth (GWD, High = 11.2 m; Mid = 10.3 m; Low = 7.1 m) between January 2011 and July 2014. The insertions (**a**; predawn water potential), (**b**; mid-day water potential), and (**d**; weighted mean soil water content) represent the mean ± 1 S.E. for each variable at each GWD (*n* = 32) when there was no significant GWD × measuring date interaction (*p* > 0.05). Different letters indicate significant differences according LSD-Fisher *post-hoc* analysis.

The groups of trees of the GWD zones were significantly different in pre-dawn (Ψ_pd_; *P* = 0.007) and noon water potential (Ψ_md_; *P* = 0.0176; Figures 1A,B, respectively). The measuring date factor and interaction between GWD × measuring date were not significant (*P* > 0.05). The mean Low-GWD Ψ_pd_ was higher than the mean Mid-GWD Ψ_pd_ and mean High-GWD Ψ_pd_ (Figure 1A). This difference was also observed among the water potential values at mid-day (Figure 1B). The GWD had no significant effect (*P* > 0.05) on the stomatal resistance (*r*_*s*_) but the measuring date was significant (*P* = 0.001) with high *r*_*s*_-values in January 2011 which decreased from July 2011 onwards (Figure 1C). The mean soil water content (WWC, mean weighted by soil depth) of the Low-GWD zone was higher than the mean water content of the Mid-GWD and High-GWD zones (Figure 1D).

**Figure 2:**
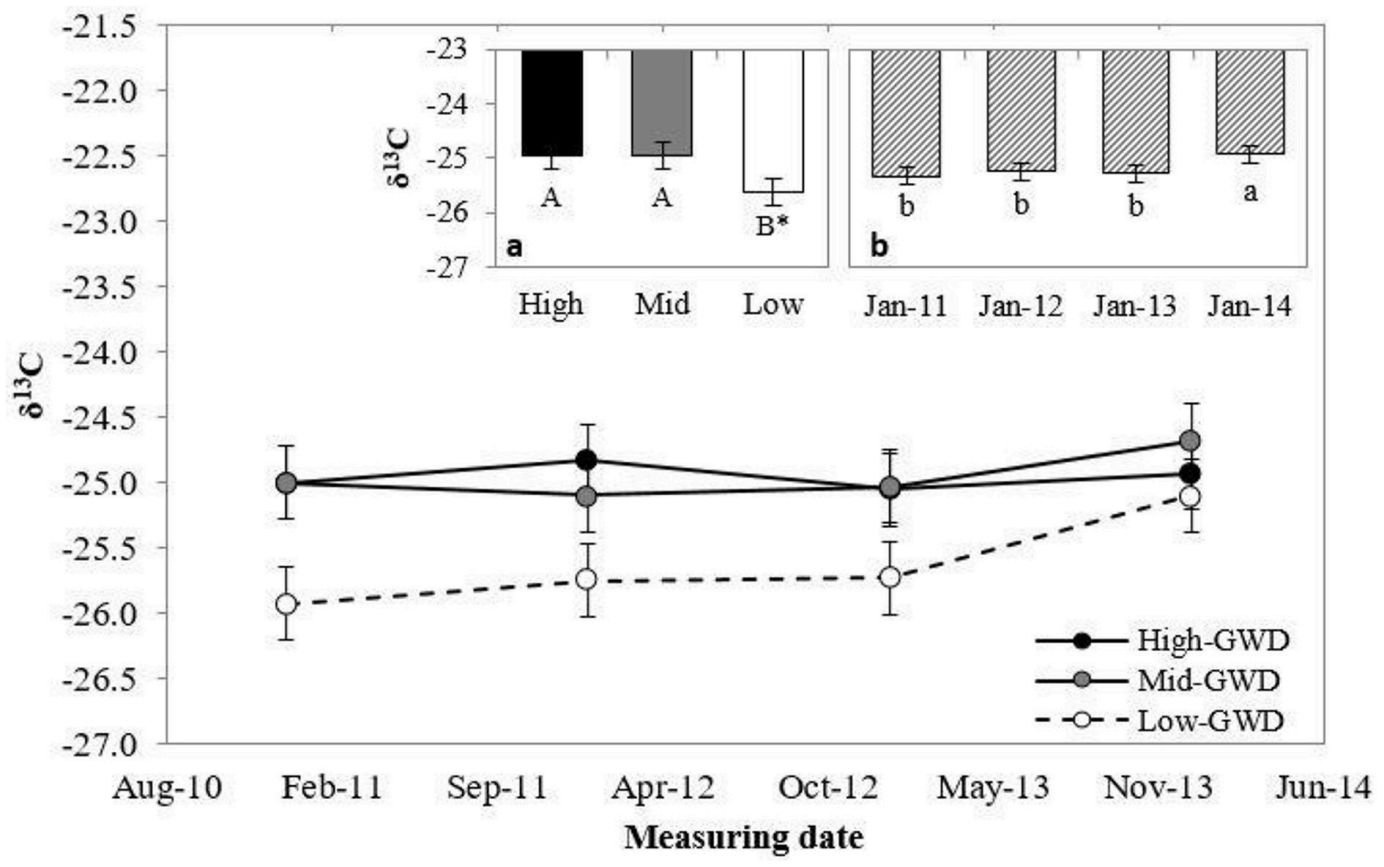
**Means ± 1 S.E. of isotopic composition of ^13^C (δ^13^C) measured in January from 2011 through 2014 in foliar samples of *P. tamarugo* trees growing at three GWDs (High, Mid, and Low as in Figure 1)**. The insertions **(a)** and **(b)** show the mean values ± 1 S.E. of δ^13^C at each GWD (*n* = 16) and at each measuring date (*n* = 12), respectively. Different letters indicate significant differences according LSD–Fisher *post-hoc* analysis. ^*^Significant difference at 10% level.

The leaf δ^13^C (Figure 2) differed between GWDs at 10% level (*P* = 0.10), with lower values in the Low-GWD zone respect to the Mid-GWD and High-GWD zones (Figure 2a) and between measuring dates (*P* = 0.0053; Figure 2b). No significant interaction between GWD and measuring date (*P* > 0.05) was observed for δ^13^C.

**Figure 3:**
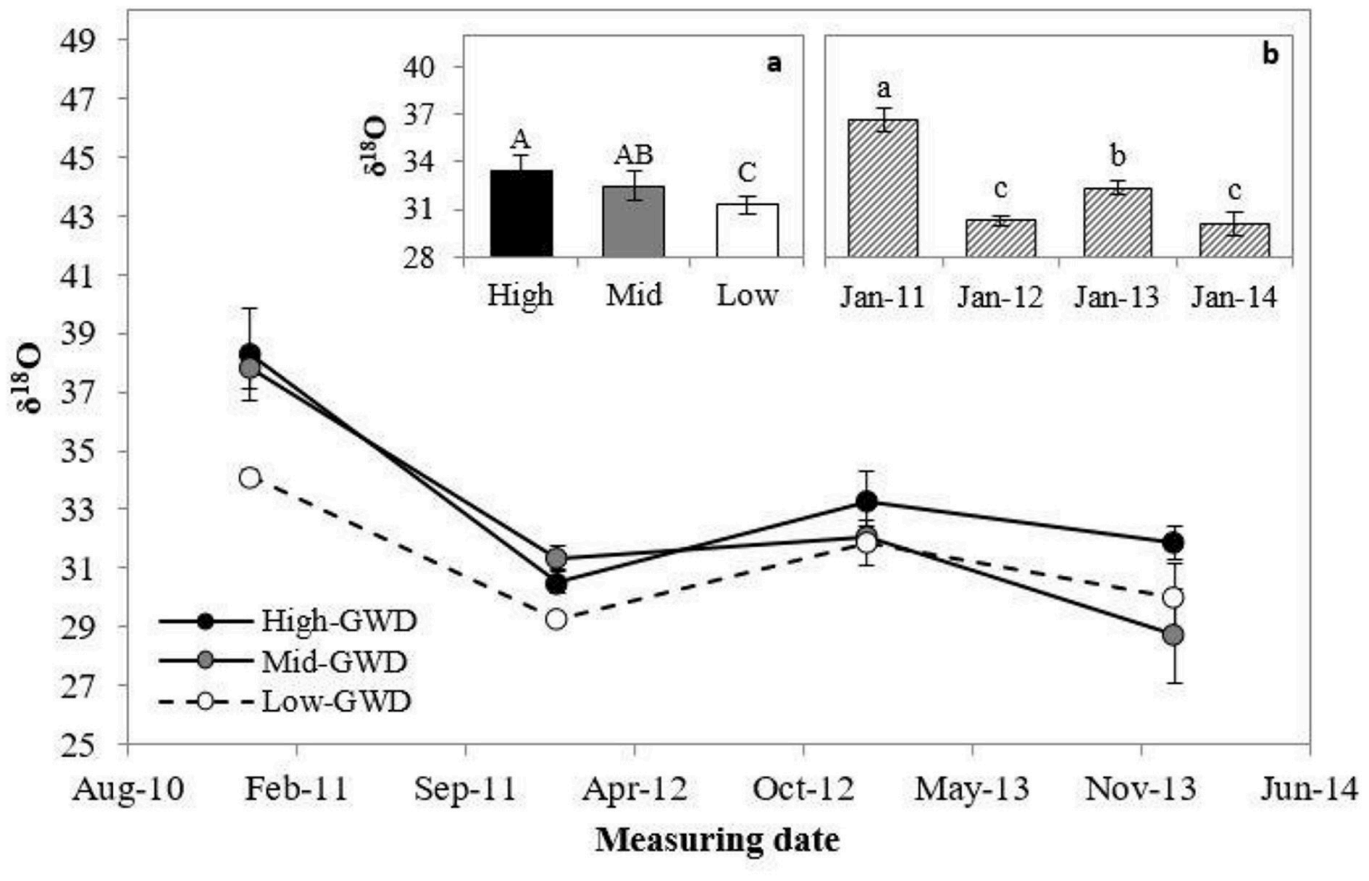
**Mean of leaf isotopic ^18^O composition ± 1 S.E. (δ^18^O) measured between January 2011 and January 2014 in *P. tamarugo* groups (*n* = 4) located at three GWDs (High, Mid, and Low as in Figure 1)**. The insertions **(a)** and **(b)** show the mean ± 1 S.E. of δ^18^O at each GWD (*n* = 16) and at each measuring date (*n* = 12), respectively. Different letters indicate significant differences according LSD–Fisher *post-hoc* analysis.

The leaf δ^18^O (Figure 3) was significantly different among GWDs (*P* = 0.0206) and between measuring dates (*P* < 0.0001). The interaction GWD x measuring date was not significant (*P* > 0.05). Figure 3a shows that the High-GWD trees had a higher value of leaf δ^18^O than the Low-GWD trees, while the Mid-GWD trees had an intermediate value equal to the leaf δ^18^O of the trees of the other two depths of the water table. Regarding the measuring date (Figure 3b), a marked seasonality was observed such that leaf δ^18^O decreased significantly from January 2011 to January 2012 repeating the cycle in 2013 and 2014 (a similar oscilation was observed in the mean relative humidity for January from 2011 through 2014, Table 1, what may indicate changes in the atmosfetric demand in January across these years). The high value of leaf δ^18^O measured in January 2011 was consistent with the high r_s_ values measured on the same date (Figure 1C).

### Effect of the water table level on the green cover and defoliation of *P. tamarugo*

**Figure 4:**
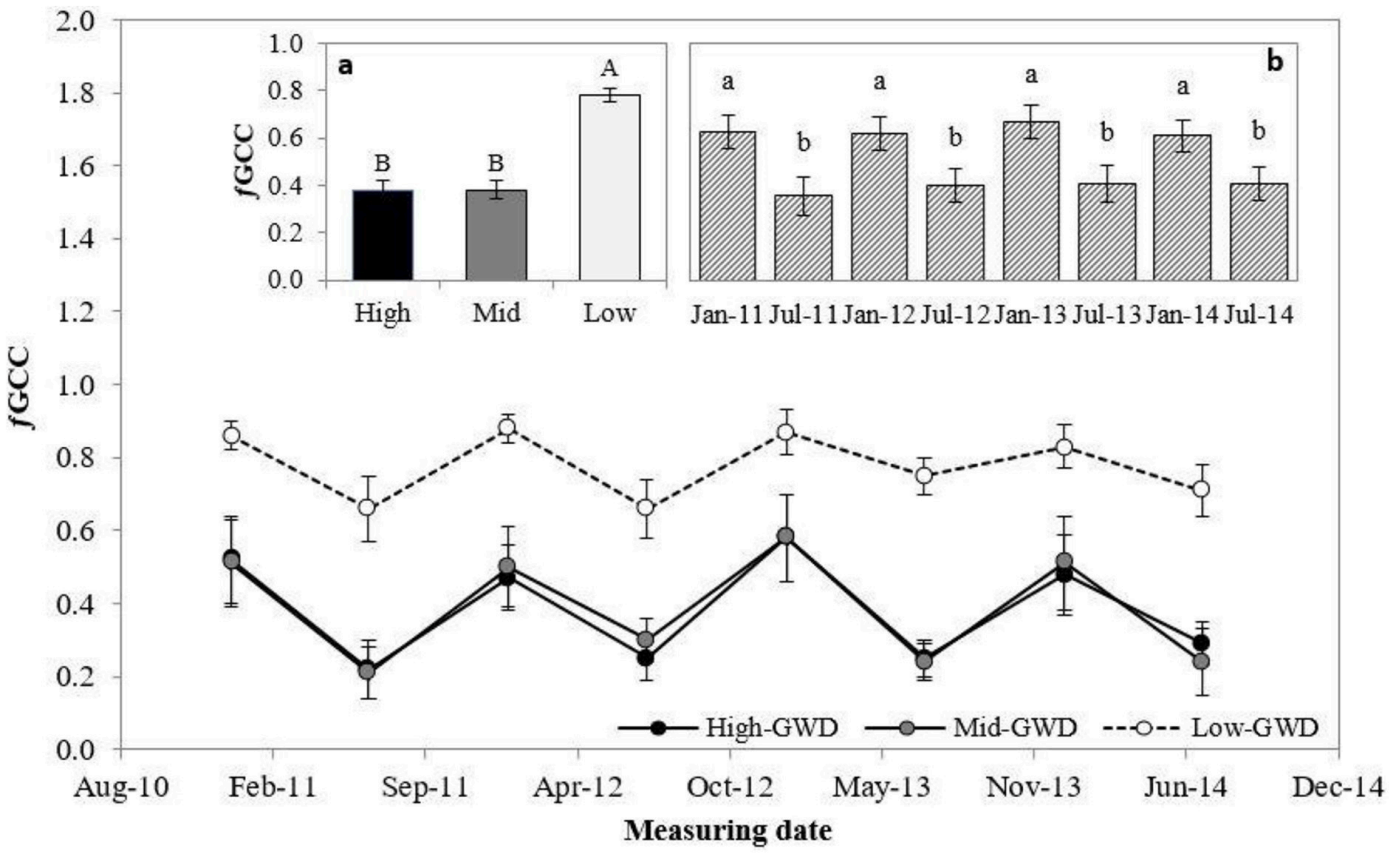
**Means ± 1 S.E. of green canopy fraction (ƒGCC) for three GWDs**. Data obtained in January and July from 2011 through 2014 (*n* = 4). The insertions **(a)** and **(b)** show the mean ƒGCC ± 1 S.E. at each GWD (*n* = 32) (High, Mid, and Low as in Figure 1) and measuring date (*n* = 12), respectively. Different letters indicate significant differences according LSD–Fisher *post-hoc* analysis.

The green cover fraction (ƒGCC; Figure 4) was significantly different between the trees at the various GWDs (*P* < 0.001) and between measuring dates (*P* < 0.001). The interaction GWD × measuring date was not significant (*P* > 0.05). Figure 4a shows that the average ƒGCC of the trees in the Low-GWD zone was significantly higher than in the trees of the Mid-GWD and High-GWD zones, which were equal among them. The mean ƒGCC of *P. tamarugo* in January of each year was significantly higher than in July in all years (Figure 4b).

The relative difference of ƒGCC between January and July (RƒGCC_Jan−Jul_) was significantly different between GWDs (*P* < 0.0001) and between measuring dates (*P* = 0.0227). The interaction GWD x measuring date was not significant (*P* > 0.05). The Mid-GWD and High-GWD trees had equal ƒGCC and had RƒGCC_Jan−Jul_ values significantly higher than the trees of the Low-GWD zone (Table 3), therefore, *P. tamarugo* trees subject to greater depths of water table, besides having a lower green coverage over time, lost a higher proportion of their foliage during the winter, however they recovered the green canopy fraction in summer.

**Table 3:** **Mean values of the relative difference between January and July green canopy fraction (RƒGCC_Jan-Jul_) at each GWD and date, significance of the factors and their interaction**.

	**R_ƒGCCJan-Jul_**	
**GWD ZONE**		
High-GWD	0.51	A
Mid-GWD	0.53	A
Low-GWD	0.20	B
**MEASURING DATE**		
2011	0.50	A
2012	0.38	B
2013	0.42	AB
2014	0.35	B
**Factors**	***p*****-values**	
GWD zone	< 0.0001	
Measuring date	0.0405	
Interaction	0.1509	

Different letters indicate significant differences according LSD-Fisher post-hoc analysis.

## Discussion

### Water relations of *P. tamarugo* in different scenarios of water table depth

The water table depth values given in Table 2 as well as the values of the physiological variables measured in this study indicate that the trees were acclimated at each GWD (there was practically no change of the variables from 2011 through 2014 at each GWD). Furthermore, the results of this study demonstrate that the GWD affects the water status of *P. tamarugo*. When the GWD was 7.1 m the Ψ_pd_ and Ψ_md_ were higher compared to a GWD of 10.3 or 11.2 m (Figures 1A,B). The differences are consistent with those reported by Chávez et al. (2013b), where at a water table depth of 11 m (Pintados Salt Flat) the trees had significantly lower Ψ_pd_ compared to a water table depth of 6 m (Llamara Salt Flat).

The minimum water potential values observed were −2.7 and −3.0 MPa for GWDs of 7.1 and 11.2 m, respectively. The maximum pre-dawn water potentials measured were −2.0 and −2.2 MPa, respectively, therefore Ψ decreased throughout the day in a parallel way across GWD zones. *P. tamarugo* in the High-GWD zone decreased its water potential in 0.7 MPa and in the Low-GWD the decrease in water potential was 0.8 MPa. The observation of a similar twig water potential change along the day for the two GWDs plus the fact that the trees are apparently not increasing the minimum *r*_*s*_ at the leaf level (Figure 1C), point toward a relatively anisohydric stomatal behavior of *P. tamarugo* (Tardieu and Simonneau, 1988; McDowell et al., 2008, 2011). The species was able to maintain low values of *r*_*s*_ under a condition of reduced water availability. This behavior is a characteristic of species that have evolved in high aridity (Maherali et al., 2004).

While instant measurements of *r*_*s*_ indicated that there were no differences between trees across the various GWDs, the foliar isotopic composition (an integrated value over time) indicated otherwise. The trees growing in the High-GWD zone had a higher mean value of δ^13^C (Figure 2a) and a higher mean δ^18^O (Figure 3a) compared to the trees growing in the Low-GWD zone. These results indicate that under increased GWD, *P. tamarugo* has lower assimilation (higher δ^13^C isotopic composition) and a higher integrated *r*_*s*_, indicated by higher values of ^18^O isotopic composition at the leaf level (Hasselquist et al., 2010).

The high value of leaf δ^18^O measured in January 2011 was consistent with the high *r*_*s*_-values measured on the same date (Figure 1C). We attribute the values of ^18^O isotopic composition of the leaf tissue to leaf transpiration, controlled by the stomatal resistance as the ^18^O isotopic composition of the groundwater of the Llamara Salt Flat is homogeneous at the GWDs considered in this study. Indeed, the ^18^O composition is not correlated with GWD (*r* = 0.072, *P* = 0.81), with mean ^18^O composition values of −6.0‰, *SD* = 0.7, *n* = 13 (Dirección General de Aguas DGA, 1985). In 2012, water samples were taken from two observation wells to measure δ^18^O, one close to the High, and the other to the Low GWD zone; the values obtained were −6.10 and −6.09‰, respectively (unpublished data, personal communication, Geohidrologia Consultores, ARCADIS, Chile).

The results suggest that *P. tamarugo* exerts control over its leaf resistance when the GWD is beyond 10.0 m and it is probable that twig Ψ values of −3.0 MPa measured under this condition could be near the threshold of loss of hydraulic functionality. It is necessary, however, to consider that these thresholds tend to be variable throughout the growing season (Kolb and Sperry, 1999), and further research is required to fully assess this point.

### Water use strategy of *P. tamarugo* growing at increased water table depths

Mooney et al. (1980) proposed that during the night, when stomatal resistance of *P. tamarugo* is maximum, the water flow in the soil-plant-atmosphere occurs from the water table through the deep roots into the plant, and from the shallow root mass into the upper part of the soil, following a strong water potential gradient generated by soil salinity and tree transpiration of the previous day. During the day, the water flow would occur from the water table and the soil surrounding the shallow root mass, to the atmosphere through the plant. Aravena and Acevedo (1985) demonstrated that the water surrounding the shallow root mass and the water present in the tree had the same isotopic composition (^18^O and ^2^H) than the water of the ground water. Figure 1D shows that the soil water content in the soil root mass zone decreased with increasing GWD. The water depletion in the soil root mass zone at higher GWDs indicates that the water flow through the deep roots of *P. tamarugo* has decreased affecting the tree water balance via decreased water supply.

At the same time *P. tamarugo* regulates its water demand via partial stomatal closure (*r*_*s*_ at the time of maximum stomatal opening did not decrease but ^18^O isotopic composition increased) and essentially through foliage loss, as has been described in other phreatophytes (Cooper et al., 2003, 2006). Studies done in young *P. tamarugo* plants subject to water stress showed an accelerated senescence (Chávez et al., 2013a), and adult plants growing in the Pintados Salt Flat (close to our study area) experienced decreased activity measured by NDVI, reaching minimum values at 10 m of GWD (Ortiz et al., 2012). A similar response has been observed in *Populus deltoides* (Cooper et al., 2003), *Populus fremontii, Salix gooddingii*, and *Tamarix chinensis* (Horton et al., 2001) when these trees face a water table drop. In this study, GWDs from 7.1 to 10.3 m decreased the mean fraction of green canopy cover from 78 to 38% (Figure 4a). Furthermore, the foliage loss in winter increased from 20 to 50% when the GWD increased from 7.1 to 10.3 m. Besides the green cover loss in winter, increased under water stress, *P. tamarugo* recovered its green canopy fraction during the summer period (Table 3), supporting an acclimation hypothesis. The decrease in green cover during winter had been already described (Acevedo et al., 2007) but the increase in seasonal foliage loss due to water stress had not been reported. The lowest values of twig water potential measured in this study were around −3.0 MPa in the *P. tamarugo* trees of the High-GWD zone. It is probable that, with this value of water potential, the trees lose a significant fraction of their hydraulic functionality, what would be consistent with the observations of ƒGCC since the foliage loss is due to a loss of hydraulic conductivity in anisohydric species experiencing low water potentials as a result of high stomatal conductance under stress conditions (Hoffmann et al., 2011). In this study we observed that in the summer periods the ƒGCC of the trees was 0.86 under Low-GWD, 0.53 under Mid-GWD, and 0.51 in the High-GWD zone, while in the winter months the values were 0.69 for the trees of the Low-GWD, 0.24 for those of the the Mid-GWD and 0.25 in High-GWD zone. While this strategy may be useful to tolerate prolonged periods of water stress under the conditions of the Atacama Desert, it also could have a disadvantage given the high intensities of radiation, temperature, and wind speed prevailing in this ecosystem. Low coverage implies that a larger fraction of the tree leaf area is exposed to direct radiation, increasing the likelihood of photoinhibition, and oxidative stress (Delatorre et al., 2008). Chávez et al. (2013a) and Chávez et al. (2014) described the mechanism of paraheliotropism in *P. tamarugo* as an avoiding mechanism of high radiation stress. They also found that paraheliotropism decreased in summer, when water stress was higher. On the other hand, lower leaf coverage increases the turbulence inside of the cup of *P. tamarugo*, decreasing the aerodynamic resistance, what would increase the rate of transpiration, inducing stomatal closure. This situation is reflected in the observed values of δ^13^C and δ^18^O composition when GWD was beyond 10 m.

The fact that each GWD was constant from 2011 through 2014 (Table 2) allow us to infer what will happen to *P. tamarugo* growing in the Llamara Salt Flat as GWD increases beyond 10 to 11 m. There will be a decrease in twig water potential, an increased integrated stomatal resistance over time (increased δ^18^O composition), a lower photosynthetic rate (increased δ^13^C) and a decreased green canopy fraction (*f* GCC). Notably, these variables changed with mean GWD but did not interact with measuring date during the 4 years of this study.

The increasing scarcity of water as a resource, particularly in arid and desert zones, will force an increased water extraction of water which will exacerbate the effect of the stressful conditions of the Atacama Desert. This will have a negative impact on the sustainability of the *P.tamarugo* ecosystem, therefore research is needed to better characterize the phenotypic plasticity of this phreatophyte in response to changes in the groundwater regime.

The balance achieved between water supply and demand was reflected in the low variation of the water potential and of the variables related to gas exchange over time for a given GWD. This acclimation capacity of *P. tamarugo* after experiencing increases in GWD has great value for the implementation of conservation strategies. Further research is needed, however, to determine the limits of the acclimation.

Even though, we are not yet in a position to definitely establish a GWD beyond which water extraction from the Tamarugal basin should be completely stopped, the physiological information reported here clearly indicates that special care should be taken when the aquifer reaches 11 m deep.

An additional observation pointing toward a *P. tamarugo* conservation strategy relates to the soil water content in the upper 1.0 m of soil, where the *P. tamarugo* root mass lies. If the *P. tamarugo* tap roots are providing water in appropropriate amount to the tree, the soil water content of this zone should not change significantly over periods of several days; on the contrary, if the *P. tamarugo* tap roots are not providing enough water to fulfill the water demand of the tree, a decrease in the soil water content of this zone, as the one reported in this article should be observed. A word of caution should be expressed here, to remind the reader that the water flux from the ground water to the soil root mass zone through the tap roots of the tree occurs along a water potential gradient, therefore, the soil salinity in the soil root mass zone will have an important influence in the water flux from the ground water to the soil of the root mass zone via tap roots.

## Conclusions

The differences observed in the physiological behavior of *P. tamarugo* trees, attributable to the ground water depth; show that increasing the depth of the water table from 7 to 11 m significantly affects the water status of *P. tamarugo*.

*P. tamarugo* has a strategy of foliage loss to balance the water demand against a diminished water supply, a characteristic of a relative anisohydric species.

At present and for conservation purposes we advise to monitor the green canopy fraction of the trees as an indicator of their status. This is an easy to measure, very sensitive variable whose maximum and minimum values were maintained during the summer and winter across several years (2011 through 2014) at 11.2 m GWD. A disruption of the equilibrium, detected by increased defoliation beyond the values reported in this paper, would indicate the need to decrease or even stop water extraction to avoid irreversible damage to the trees. In the meantime, additional information should be gathered to tune-up this recommendation.

## Author contributions

MG collected data and drafted this manuscript. PS provided logistic support, participated in designing the experiment and edited the manuscript. EA designed the experiment, followed up on data collection, provided support through the Soil-Water Relations Laboratory and edited the manuscript.

## Funding

The funding sources of the Soil-Plant_Water Relations Laboratory are: The University of Chile, FONDECYT, CONICYT, SQM, FIA.

### Conflict of interest statement

The authors declare that the research was conducted in the absence of any commercial or financial relationships that could be construed as a potential conflict of interest. The Soil-Plant-Water Relations Laboratory of the University of Chile has an agreement with SQM, a Chilean Mining Company under which it monitors the water relations of *Prosopis tamarugo* Phil. This is a requirement that the Chilean Environmental Authority imposes on SQM to allow ground water pumping from the Llamara Salt Flat. This research has been partially financed by this agreement.
