# Supplementary material for: Water Relations and Foliar Isotopic Composition of Prosopis tamarugo Phil., an Endemic Tree of the Atacama Desert Growing at Three Levels of Water Table Depth
Source: Front Plant Sci. 2016 Mar 30;7:375. doi: 10.3389/fpls.2016.00375 (PMC4811898; doi:10.3389/fpls.2016.00375)
Supplement: Supplementary file 1 [file Table1.docx]

Supplementary Material

Water relations and foliar isotopic composition of *Prosopis tamarugo* Phil., an endemic tree of the Atacama Desert growing under three levels of water table depth.

Marco Garrido^1^, Paola Silva^2^, Edmundo Acevedo^2^*

^1^ Programa de Doctorado en Ciencias Silvoagropecuarias y Veterinarias, University of Chile, Santiago, Chile,

^2^ Soil-Plant-Water Relations Laboratory, Agricultural Production Department, Faculty of Agronomical Sciences, University of Chile, Santiago, Chile

*** Correspondence:** Edmundo Acevedo, University of Chile, Soil-Plant-Water Relations Laboratory, Casilla 1004, Santiago, Chile.

[eacevedo@u.uchile.cl](mailto:eacevedo@u.uchile.cl)

**Supplementary Table 1.** Isotopic composition of leaf tissue δ^13^C and δ18O of *Prosopis tamarugo* growing at three groundwater depths (Low=7.1m;Midium=10.0m ; High= 11.7m). The Low GWD is a reference depth at which physiological processes are not affected. Data are provided for each year and Measuring Date (January).

| Measuring date | GWD | Tree | δ^13^C (‰) | δ^18^O (‰) |
| --- | --- | --- | --- | --- |
| Jan-11 | High | A01 | -25.1 | 36.5 |
| Jan-11 | High | A02 | -24.2 | 42.6 |
| Jan-11 | High | A17 | -25.5 | 38.6 |
| Jan-11 | High | A19 | -25.2 | 35.4 |
| Jan-12 | High | A01 | -25.3 | 29.7 |
| Jan-12 | High | A02 | -23.7 | 30.4 |
| Jan-12 | High | A17 | -25.2 | 31.5 |
| Jan-12 | High | A19 | -25.1 | 30.4 |
| Jan-13 | High | A01 | -25.5 | 31.5 |
| Jan-13 | High | A02 | -23.9 | 31.9 |
| Jan-13 | High | A17 | -25.2 | 36 |
| Jan-13 | High | A19 | -25.6 | 33.7 |
| Jan-14 | High | A01 | -25.1 | 30.1 |
| Jan-14 | High | A02 | -24.1 | 32.4 |
| Jan-14 | High | A17 | -24.9 | 32.4 |
| Jan-14 | High | A19 | -25.6 | 32.5 |
| Jan-11 | Mid | A05 | -24.6 | 39.5 |
| Jan-11 | Mid | A15 | -25.2 | 38.4 |
| Jan-11 | Mid | A20 | -25.0 | 36.3 |
| Jan-11 | Mid | A50 | -25.2 | 37.0 |
| Jan-12 | Mid | A05 | -24.8 | 31.2 |
| Jan-12 | Mid | A15 | -25.4 | 32.2 |
| Jan-12 | Mid | A20 | -24.6 | 30.4 |
| Jan-12 | Mid | A50 | -25.6 | 31.6 |
| Jan-13 | Mid | A05 | -24.7 | 32.2 |
| Jan-13 | Mid | A15 | -25.4 | 33 |
| Jan-13 | Mid | A20 | -24.8 | 31.2 |
| Jan-13 | Mid | A50 | -25.2 | 31.9 |
| Jan-14 | Mid | A05 | -24.7 | 24.0 |
| Jan-14 | Mid | A15 | -25.3 | 29.8 |
| Jan-14 | Mid | A20 | -24.1 | 31.4 |
| Jan-14 | Mid | A50 | -24.6 | 29.5 |
| Jan-11 | Low | A38 | -25.8 | 33.8 |
| Jan-11 | Low | A41 | -26.8 | 33.8 |
| Jan-11 | Low | A43 | -25.0 | 34.8 |
| Jan-11 | Low | A44 | -26.1 | 34.0 |
| Jan-12 | Low | A38 | -26.0 | 29.2 |
| Jan-12 | Low | A41 | -26.2 | 29.3 |
| Jan-12 | Low | A43 | -25.4 | 29.4 |
| Jan-12 | Low | A44 | -25.4 | 29.1 |
| Jan-13 | Low | A38 | -25.7 | 29.9 |
| Jan-13 | Low | A41 | -26.4 | 33.6 |
| Jan-13 | Low | A43 | -25.0 | 32 |
| Jan-13 | Low | A44 | -25.8 | 31.9 |
| Jan-14 | Low | A38 | -24.7 | 31.5 |
| Jan-14 | Low | A41 | -25.9 | 31.9 |
| Jan-14 | Low | A43 | -24.7 | 29.8 |
| Jan-14 | Low | A44 | -25.1 | 26.8 |
