# Supplementary material for: Water Relations and Foliar Isotopic Composition of Prosopis tamarugo Phil., an Endemic Tree of the Atacama Desert Growing at Three Levels of Water Table Depth
Source: Front Plant Sci. 2016 Mar 30;7:375. doi: 10.3389/fpls.2016.00375 (PMC4811898; doi:10.3389/fpls.2016.00375)
Supplement: Supplementary file 2 [file Table2.docx]

Supplementary Material

Water relations and foliar isotopic composition of *Prosopis tamarugo* Phil., an endemic tree of the Atacama Desert growing under three levels of water table depth.

Marco Garrido^1^, Paola Silva^2^, Edmundo Acevedo^2^*

^1^ Programa de Doctorado en Ciencias Silvoagropecuarias y Veterinarias, University of Chile, Santiago, Chile,

^2^ Soil-Plant-Water Relations Laboratory, Agricultural Production Department, Faculty of Agronomical Sciences, University of Chile, Santiago, Chile

*** Correspondence:** Edmundo Acevedo, University of Chile, Soil-Plant-Water Relations Laboratory, Casilla 1004, Santiago, Chile.

[eacevedo@u.uchile.cl](mailto:eacevedo@u.uchile.cl)

**Supplementary Table 2. ANOVA Tables for physiological variables (Water relations and foliar isotopic composition of Prosopis tamarugo Phil. a native legume tree of the Atacama Desert growing at three levels of water table depth).**

| **Predawn water potential** | | | |
| --- | --- | --- | --- |
| **Factor** | **numDF** | **F-value** | **p-value** |
| **(Intercept)** | 1 | 2913.55 | <0.0001 |
| **Measuring date** | 7 | 1.4 | 0.2187 |
| **GWD** | 2 | 5.32 | 0.007 |
| **Measuring date:GWD** | 14 | 0.59 | 0.8679 |
|  |  |  |  |
|  |  |  |  |
| **Midday water potential** | | | |
| **Factor** | **numDF** | **F-value** | **p-value** |
| **(Intercept)** | 1 | 5059 | <0.0001 |
| **Measuring date** | 7 | 0.44 | 0.874 |
| **GWD** | 1 | 6.04 | 0.0176 |
| **Measuring date:GWD** | 7 | 0.36 | 0.9221 |
|  |  |  |  |
|  |  |  |  |
| **Stomatal resistance** | | | |
| **Factor** | **numDF** | **F-value** | **p-value** |
| **(Intercept)** | 1 | 3111.48 | <0.0001 |
| **Measuring date** | 7 | 18.5 | <0.0001 |
| **GWD** | 2 | 2.3 | 0.1078 |
| **Measuring date:GWD** | 14 | 1.53 | 0.1223 |
|  |  |  |  |
|  |  |  |  |
| **Leaf δ^13^C** | | | |
| **Factor** | **numDF** | **F-value** | **p-value** |
| **(Intercept)** | 1 | 29849.3 | <0.0001 |
| **Measuring date** | 3 | 5 | 0.0053 |
| **GWD** | 2 | 2.38 | 0.1006 |
| **Measuring date:GWD** | 6 | 1.35 | 0.2623 |
|  |  |  |  |
|  |  |  |  |
| **Leaf δ^18^O** | | | |
| **Factor** | **numDF** | **F-value** | **p-value** |
| **(Intercept)** | 1 | 11530.14 | <0.0001 |
| **Measuring date** | 3 | 41.76 | <0.0001 |
| **GWD** | 2 | 4.33 | 0.0206 |
| **Measuring date:GWD** | 6 | 1.97 | 0.0959 |
|  |  |  |  |
|  |  |  |  |
| ***f*GCC** | | | |
| **Factor** | **numDF** | **F-value** | **p-value** |
| **(Intercept)** | 1 | 224.42 | <0.0001 |
| **Measuring date** | 7 | 34.59 | <0.0001 |
| **GWD** | 2 | 14.56 | <0.0001 |
| **Measuring date:GWD** | 14 | 1.12 | 0.3526 |
|  |  |  |  |
|  |  |  |  |
| **Relative difference of *f*GCC** | | | |
| **Factor** | **numDF** | **F-value** | **p-value** |
| **(Intercept)** | 1 | 364.43 | <0.0001 |
| **Measuring date** | 3 | 3.6 | 0.0227 |
| **GWD** | 2 | 25.54 | <0.0001 |
| **Measuring date:GWD** | 6 | 2.06 | 0.0822 |
